# Application of a Biosurfactant from *Bacillus velezensis* GJ1 as an Emulsifier and Antibacterial Agent for Food Systems

**DOI:** 10.4014/jmb.2603.03037

**Published:** 2026-05-12

**Authors:** Geum-Jae Jeong, Younggwon On, Hyo-Jin Kim, Do-Kyun Kim, Na-Yeon Kim, Chae-Won Baek, Ye-Hyeon Jo, Min-Ung Kim, Minji Kim, Kyung-Jin Cho, Young-Mog Kim

**Affiliations:** 1 Department of Food Science and Technology, Pukyong National University, Busan 48513, Republic of Korea; 2 Marine Integrated Biomedical Technology Center, The National Key Research Institutes in Universities, Pukyong National University, Busan 48513, Republic of Korea; 3 Research Center for Marine Integrated Bionics Technology, Pukyong National University, Busan 48513, Republic of Korea; 4 Division of Antimicrobial Resistance Research, Korea National Institute of Health, Korea Disease Control and Prevention Agency, Cheongju 28159, Republic of Korea; 5 Interdisciplinary Program of Blue Food, Pukyong National University, Busan 48513, Republic of Korea; 6 Korea Food Research Institute, Wanju 55365, Republic of Korea

**Keywords:** Biosurfactant, *Bacillus velezensis*, Whole-genome sequencing, Emulsification, Antibacterial activity, Food systems

## Abstract

The demand for natural and multifunctional ingredients in food systems has increased interest in microbial biosurfactants as potential alternatives to synthetic emulsifiers and antimicrobial agents. However, their practical functionality in food systems remains insufficiently explored. Here, a biosurfactant produced by *Bacillus velezensis* GJ1 was evaluated as a functional emulsifier and antibacterial agent for food applications. Whole-genome sequencing confirmed the presence of lipopeptide biosynthetic gene clusters associated with surfactin and iturin production, while no genes related to known virulence factors were detected, supporting the safety of the strain. The emulsifying performance of the biosurfactant was assessed using various food-grade oils and compared with commercial surfactants. The biosurfactant formed stable emulsions with canola, corn, grapeseed, olive, soybean, and sunflower oils, exhibiting emulsification indices comparable to those of Tween 80 and Span 80. Microstructural analysis further revealed that emulsions stabilized with the biosurfactant displayed droplet characteristics similar to those formed with Tween 80. In addition to its emulsifying functionality, the biosurfactant exhibited antibacterial activity against *Escherichia coli*, with minimum inhibitory and bactericidal concentrations of 256 and 512 μg/mL, respectively, comparable to those of the food-grade antimicrobial nisin. Application in a sausage-based food model resulted in a significant reduction of *E. coli* populations, achieving a maximum decrease of approximately 2.8 log CFU/g and exceeding the antibacterial effect of nisin under the same conditions. These findings demonstrate that the biosurfactant produced by *B. velezensis* GJ1 exhibits both emulsifying and antibacterial functionalities, highlighting its potential as a natural multifunctional ingredient for food systems.

## Introduction

Biosurfactants are surface-active compounds produced by microorganisms that reduce surface and interfacial tension and have attracted considerable interest due to their biodegradability, low toxicity, and functional versatility [1]. These compounds have been explored for applications in diverse fields such as environmental remediation [2], pharmaceuticals [3], cosmetics [4], and agriculture [5]. More recently, their potential for food-related applications has gained increasing attention as the demand for natural and sustainable functional ingredients continues to increase [6]. Among microbial biosurfactants, lipopeptides produced by *Bacillus* species, such as surfactin, have been extensively studied due to their strong surface activity and multifunctional properties [1, 7, 8]. In particular, the biosurfactant produced by *B. velezensis* GJ1 has been reported to consist predominantly of surfactin as a major component and to exhibit notable surface-active properties along with antimicrobial activity against pathogenic bacteria [9]. Importantly, *B. velezensis* is listed as a food-approved microorganism in the Korean Food Standards Codex [10], indicating that metabolites derived from this species may be suitable for food applications. Together, these characteristics suggest that biosurfactants produced by *B. velezensis* GJ1 may serve as promising multifunctional ingredients for food applications.

Many food products are inherently complex systems composed of immiscible phases, most commonly oil and water, which often require appropriate physical stabilization to achieve desirable quality attributes [11]. Emulsification enables the formation of dispersed oil droplets within an aqueous phase and contributes significantly to determining the texture, appearance, and shelf life of a wide range of foods, including sauces, dressings, beverages, and processed meat products [12]. The stability of food emulsions is influenced not only by the ability to form emulsions initially but also by the resistance of dispersed droplets to coalescence, creaming, and phase separation during storage [12]. These stability characteristics are closely associated with the size, shape, and spatial distribution of emulsion droplets, which collectively define the colloidal structure of the system [13]. In food systems, emulsifier performance is therefore closely linked to emulsion stability during storage and the physical characteristics of dispersed oil droplets. Accordingly, evaluation of emulsification performance in food applications can benefit from consideration of both emulsion stability and droplet-level characteristics.

In addition to their surface-active properties, biosurfactants are known to exhibit antimicrobial activity through multiple mechanisms, including disruption of microbial cell membranes, alteration of membrane permeability, and interference with microbial adhesion to surfaces [9, 14, 15]. Their amphiphilic nature makes biosurfactants especially attractive for antimicrobial applications in food systems, where interactions with both lipid and aqueous phases are critical [16]. When applied in food systems, however, their antimicrobial behavior may be influenced by interactions with surrounding food components. The heterogeneous nature of food matrices, together with the presence of lipids and proteins, can affect the distribution and local concentration of antimicrobial agents at microbial targets [17]. Consequently, evaluation of biosurfactant efficacy in food applications benefits from consideration of both antimicrobial mechanisms and practical performance within food matrices.

Previous studies on biosurfactants produced by *B. velezensis* GJ1 have primarily focused on antibacterial mechanisms, whereas their applicability in food systems has remained largely unexplored. Therefore, the present study aimed to evaluate the potential of a *B. velezensis* GJ1-derived biosurfactant specifically for food-related applications. Whole-genome sequencing (WGS) was performed to provide genetic support for biosurfactant production and to assess the absence of safety-related factors relevant to food use. The emulsifying performance of the biosurfactant was investigated using food-grade oils and comparatively evaluated against commonly used food-grade surfactants, Tween 80 and Span 80, with an emphasis on emulsion stability and microstructural characteristics. In parallel, its antibacterial activity was assessed in comparison with nisin, a representative food-grade antimicrobial, both *in vitro* against a foodborne pathogen and in a sausage-based food matrix. Through this integrated genomic and functional approach, this study seeks to advance the understanding of *B. velezensis* GJ1-derived biosurfactants as multifunctional ingredients for food systems.

## Materials and Methods

### Materials

Chloroform, hydrochloric acid, methanol, nisin, sodium hydroxide, Span 80, and Tween 80 were purchased from Sigma-Aldrich (USA). Canola oil, corn oil, grapeseed oil, olive oil, soybean oil, and sunflower oil were purchased from a local market (Republic of Korea). Mueller-Hinton agar (MHA), Mueller-Hinton broth (MHB), tryptic soy agar (TSA), and tryptic soy broth (TSB) were obtained from BD Difco (USA). Phosphate-buffered saline (PBS) was purchased from Thermo Fisher Scientific (USA). All solvents used in this study were of analytical grade.

### Microorganism

*B. velezensis* GJ1, previously isolated by [9], was used for biosurfactant production. The strain was taxonomically identified through 16S rRNA gene sequence analysis, and the 16S rRNA gene sequence was deposited in the GenBank database under the accession number PV785483. *Escherichia coli* (1682), used as a representative Gram-negative bacterium and a commonly employed indicator organism for food hygiene and contamination [18], was obtained from the Korean Collection for Type Cultures (Republic of Korea). All bacterial strains were cultured on TSA at 37°C and subcultured at two-week intervals to maintain viability.

### Whole-Genome Sequence and Annotation

WGS of *B. velezensis* GJ1 was performed by a commercial sequencing service provider (Macrogen, Republic of Korea). The genome was sequenced using a hybrid sequencing strategy combining short-read sequencing on the Illumina platform (USA) and long-read sequencing on the Oxford Nanopore Technologies platform (UK). Hybrid genome assembly was conducted using Unicycler (v0.5.1) with default parameters to generate the final assembled genome. Genome annotation was subsequently performed using Prokka with bacterial annotation settings. Basic genomic features, including genome size, GC content, number of predicted genes, protein-coding sequences, rRNAs, and tRNAs, were extracted from the assembled and annotated genome. To identify genes associated with biosurfactant biosynthesis, predicted protein sequences were screened for homologs of previously reported biosynthetic operons. Gene presence was determined based on sequence similarity to reference genes. In addition, the assembled genome was screened against the Virulence Factor Database (VFDB) to assess the presence of genes associated with known virulence factors. The whole genome shotgun project of *B. velezensis* GJ1 has been deposited at DDBJ/ENA/GenBank under the accession number JBVUOD000000000.

### Biosurfactant Production and Recovery

Biosurfactant production and recovery were conducted following the protocol reported by [14]. The bacterial strain was cultivated in TSB at 37°C with continuous shaking at 150 rpm for 5 days to allow extracellular biosurfactant accumulation. At the end of the cultivation period, the broth was subjected to centrifugation (27,000 × *g*, 20 min) to pellet the bacterial biomass. The cell-free supernatant was then clarified by filtration using a 0.2 μm membrane filter. The clarified supernatant was adjusted to pH 2.0 by the gradual addition of 6 M hydrochloric acid under gentle agitation to induce biosurfactant precipitation. The acidified solution was maintained under static conditions for approximately 16 h, after which the precipitated material was collected by centrifugation at 27,000 × *g* for 20 min. The recovered precipitate was resuspended in deionized water, and the pH was subsequently adjusted to 7.0 using 1 M sodium hydroxide. Biosurfactant extraction was then performed using organic solvent partitioning. A chloroform-methanol mixture (2:1, *V/V*) was added to the neutralized aqueous solution to achieve a final solvent ratio of chloroform:methanol:water = 8:4:3 (*V/V/V*). The biphasic system was agitated overnight to facilitate transfer of biosurfactant molecules into the organic phase. Following phase separation, the organic layer was carefully collected and concentrated by solvent evaporation under reduced pressure at 25°C using a rotary evaporator. The evaporated extract was placed at -80°C and subsequently subjected to lyophilization for 96 h to obtain crude biosurfactant as a dry powder. The dried biosurfactant was stored at -20°C until further use. The chemical composition of the GJ1-derived biosurfactant has been previously characterized, with surfactin C identified as the predominant component [9].

### Evaluation of Emulsifying Activity against Food-Grade Oils

The emulsifying activity of the *B. velezensis* GJ1-derived biosurfactant against food-grade oils was evaluated using a modified emulsification index (EI) method, as reported previously [1]. Briefly, equal volumes (2 mL each) of biosurfactant solution and food-grade oil were transferred into glass test tubes and vigorously vortexed for 2 min to promote emulsion formation. The food-grade oils used in this study were canola oil, corn oil, grapeseed oil, olive oil, soybean oil, and sunflower oil. The biosurfactant solution was prepared at a concentration of 125 mg/L, corresponding to its critical micelle concentration (CMC) [9]. For comparison, the commercial food-grade surfactants Tween 80 and Span 80 were prepared at their respective CMCs, with Tween 80 at 24.7 mg/L [19] and Span 80 at 1.82 mmol/L [20]. The prepared emulsions were maintained at room temperature under static conditions, and the thickness of the emulsion layer was determined both immediately following formation (Day 0) and 24 h later (Day 1). The EI was calculated as the ratio of the emulsion layer height to the total height of the liquid column and expressed as a percentage. For microstructural observation, representative emulsions were collected after 24 h and visualized using an optical microscope.

### *In vitro* Antibacterial Activity against Foodborne Pathogen

The minimum inhibitory concentration (MIC) and minimum bactericidal concentration (MBC) of the *B. velezensis* GJ1-derived biosurfactant were determined against *E. coli* using a broth microdilution method based on the Clinical and Laboratory Standards Institute guidelines, with minor modifications [21]. Briefly, *E. coli* was cultured in TSB at 37°C with shaking at 150 rpm until reaching the exponential growth phase. The culture was subsequently diluted in MHB to achieve a starting inoculum of approximately 6 log CFU/mL. The biosurfactant solution was serially diluted two-fold in MHB using 96-well microplates to achieve final concentrations yielding a concentration gradient from 1 up to 2048 μg/mL. Each well received the same volume of the prepared bacterial inoculum. Nisin, a commonly used food-grade antimicrobial, was included as a reference control, while wells containing bacterial suspension without treatment served as growth controls. Plates were maintained at 37°C under static conditions for 24 h, and bacterial proliferation was subsequently quantified by recording optical density at 600 nm with a microplate reader (BioTek Instruments, USA). The MIC was defined as the lowest tested concentration producing a ≥ 90% decrease in growth compared with the growth control. To determine the MBC, 10 μL samples were taken from wells that exhibited no visible growth in the MIC assay and plated onto MHA. After incubation at 37°C for 24 h, the MBC was identified as the lowest concentration at which no colonies were recovered.

### Evaluation of Antibacterial Efficacy in a Sausage Model System

The antibacterial efficacy of the *B. velezensis* GJ1-derived biosurfactant in a sausage model system was evaluated with minor modifications of previously described methods [22, 23]. Commercial sausages were obtained from a local market in Busan, Republic of Korea, aseptically opened, and sectioned into pieces of identical dimensions (1.5 × 1.5 × 1 cm). Prior to inoculation, the sausage surfaces were exposed to ultraviolet light for 15 min under aseptic conditions. An overnight culture of *E. coli* grown in TSB was diluted and inoculated onto the sausage surface to obtain a final population of approximately 5–6 log CFU/g per sample. The inoculated samples were held at 4°C for 12 h prior to antimicrobial treatment. The samples were then treated with the biosurfactant at concentrations corresponding to 1× MBC, 2× MBC, and 4× MBC, and stored at 4°C for an additional 12 h. Nisin was included as a reference antimicrobial control, while samples treated with sterile PBS served as untreated controls. Following treatment, each sample was aseptically transferred into sterile bags containing PBS and homogenized for 3 min using a stomacher. The homogenates were serially diluted in PBS, and 100 μL aliquots of each dilution were spread onto TSA plates. After incubation at 37°C for 24 h, surviving bacterial populations were enumerated and expressed as log CFU/g.

### Statistical Analysis

Statistical analysis was conducted using GraphPad Prism (v10.6.1). Statistical comparisons among groups were performed by one-way analysis of variance, and post hoc analyses were conducted using either Tukey’s or Dunnett’s test according to the study design. Statistical differences among treatment groups are indicated by distinct letters (*p* < 0.05), whereas asterisks represent significant differences relative to the untreated control (**p < 0.01, ***p < 0.001, ****p < 0.0001). The notation “ns” denotes the absence of a statistically significant difference.

## Results and Discussion

### Genomic Features of *B. velezensis* GJ1 Related to Biosurfactant Production and Food Application

**Fig. 1:**
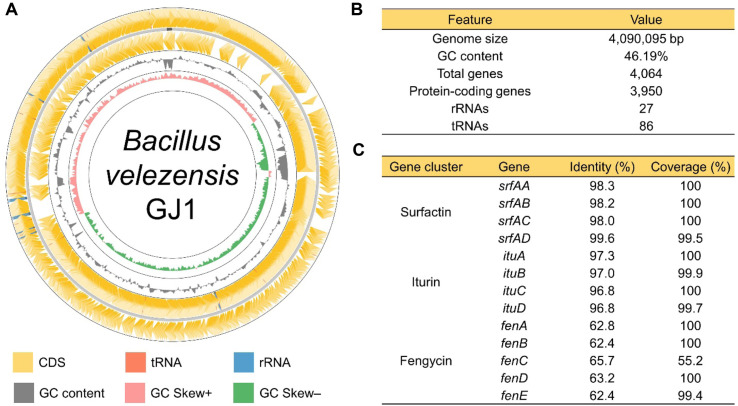
Whole-genome analysis (WGS) of *Bacillus velezensis* GJ1. (**A**) Circular genome map showing the distribution of coding sequences, tRNAs, rRNAs, and GC content across the chromosome. (**B**) Genome statistics including genome size, GC content, and numbers of predicted genes and RNA elements. (**C**) Biosynthetic gene clusters associated with lipopeptide biosurfactant production, including the surfactin (*srfAA*–*srfAD*), iturin (*ituA*–*ituD*), and fengycin (*fenA*–*fenE*) clusters identified in the *B. velezensis* GJ1 genome.

WGS was performed to characterize the genome of *B. velezensis* GJ1 and to investigate genes associated with biosurfactant production and its potential for food-related applications (Fig. 1A). The assembled genome consisted of a single circular chromosome with a total size of 4,090,095 bp and a GC content of 46.19% (Fig. 1B). Genome annotation identified 4,064 predicted genes, including 3,950 protein-coding sequences, 27 rRNA genes, and 86 tRNA genes. These genomic characteristics are consistent with previously reported genomes of *B. velezensis* strains, which typically range from 3.9 to 4.1 Mb with GC contents of approximately 46–47% [24-26]. Genome mining further revealed the presence of multiple biosynthetic gene clusters associated with lipopeptide biosurfactant production (Fig. 1C). In particular, the complete surfactin biosynthetic gene cluster (*srfAA*–*srfAD*) was detected with high sequence identity and coverage relative to previously reported surfactin synthetase genes [27]. In addition, the iturin biosynthetic gene cluster (*ituA*–*ituD*) was identified with high sequence similarity [28]. Genes associated with fengycin biosynthesis (*fenA*–*fenE*) were partially detected [29], although relatively lower sequence similarity or coverage was observed for some genes within this cluster. The presence of these non-ribosomal peptide synthetase (NRPS) gene clusters indicates that *B. velezensis* GJ1 possesses the genetic potential to produce multiple lipopeptide biosurfactants, a characteristic commonly reported in *B. velezensis* and related species [30]. These genomic findings are consistent with the lipopeptide composition previously reported for the biosurfactant produced by *B. velezensis* GJ1. In our earlier study, mass spectrometric profiling revealed the presence of surfactin-type lipopeptides, including surfactin A, surfactin C, and pumilacidin A, as well as a minor amount of iturin A [9, 31]. Pumilacidins are known members of the surfactin family of cyclic lipopeptides and are synthesized by the same NRPS gene cluster responsible for surfactin biosynthesis [32]. Therefore, the detection of the *srf* and *itu* gene clusters in the genome of *B. velezensis* GJ1 provides genetic evidence supporting the previously observed biosurfactant composition. In addition to biosurfactant biosynthesis, the genome was examined for genes associated with virulence or toxin production that could raise safety concerns for food-related applications. No genes encoding known foodborne toxins or major virulence factors were detected in the *B. velezensis* GJ1 genome. Notably, the *B. velezensis* GJ1 genome lacks complete gene clusters encoding major foodborne toxins commonly associated with the *Bacillus* genus, including hemolysin BL (*hblACD*), non-hemolytic enterotoxin (*nheABC*), and the emetic toxin cereulide (*ces*). Furthermore, analysis against the VFDB confirmed the absence of key virulence factors involved in adhesion, invasion, and intracellular survival. In addition to the genomic evidence presented in this study, previous work has reported that the biosurfactant produced by *B. velezensis* GJ1 exhibits low cytotoxicity and negligible hemolytic activity [9], suggesting its potential safety for food-related applications. This observation is consistent with previous studies reporting that many *B. velezensis* strains are considered safe and have been widely explored for diverse biotechnological applications [26, 33].

### Emulsifying Performance of the *B. velezensis* GJ1 Biosurfactant against Food-grade Oils

**Fig. 2:**
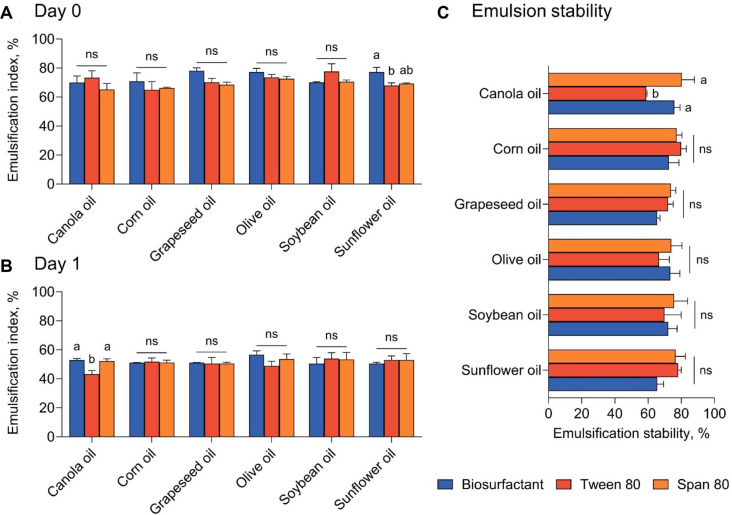
Emulsification performance of the *Bacillus velezensis* GJ1-derived biosurfactant compared with commercial surfactants. (**A**) Emulsification index (EI) of emulsions prepared with GJ1-derived biosurfactant, Tween 80, and Span 80 using various food-grade oils immediately after emulsion formation (Day 0) (*n* = 3). (**B**) EI of the same emulsions after 24 h of storage (Day 1) (*n* = 3). (**C**) Emulsion stability expressed as the percentage of EI retained from Day 0 to Day 1. Bars represent mean ± standard deviation. Different letters indicate significant differences among surfactants within the same oil (*p* < 0.05), and ns indicates no significant difference.

Emulsification between food-grade oils and aqueous phases is a key process in many food systems, as the ability to form and maintain oil-water interfaces directly influences product texture, appearance, and shelf life [34]. The emulsifying performance of the *B. velezensis* GJ1-derived biosurfactant was examined using representative food-grade vegetable oils and compared with that of commonly used commercial surfactants, Tween 80 and Span 80, based on EI values. At Day 0, biosurfactant successfully formed emulsions with all tested food-grade oils, including canola, corn, grapeseed, olive, soybean, and sunflower oils (Fig. 2A). The EI values obtained with biosurfactant were within a similar range to those observed for Tween 80 and Span 80 across the different oils, indicating effective initial emulsion formation. These results demonstrate that biosurfactant exhibits interfacial activity comparable to that of commercially available food-grade surfactants immediately after emulsion preparation. After 24 h of storage, EI values decreased for all surfactants across the tested oils (Fig. 2B), reflecting the typical evolution of oil-water emulsions over time. Importantly, biosurfactant maintained emulsification performance at levels comparable to those of Tween 80 and Span 80 for most oils, suggesting that emulsions stabilized with biosurfactant display short-term stability characteristics similar to those achieved using conventional surfactants. Oil-dependent emulsification behavior has also been reported for microbial biosurfactants, including sophorolipids produced by *Candida* spp., which exhibit variable emulsification indices depending on vegetable oil type while maintaining effective emulsifying potential across food-grade oils [35]. In line with these observations, the biosurfactant produced by *B. velezensis* GJ1 exhibited robust emulsification performance across diverse vegetable oils, supporting its functional comparability to commercial surfactants [36]. Emulsion stability, expressed as the retention of EI from Day 0 to Day 1, further supported this trend (Fig. 2C). Across all tested oils, the stability profiles of emulsions prepared with biosurfactant were similar to those obtained with Tween 80 and Span 80. The ability of GJ1-derived biosurfactant to maintain emulsification performance over time across oils with differing physicochemical properties indicates that its emulsifying behavior is not restricted to a specific oil type [37]. Overall, EI measurements show that the biosurfactant achieves emulsification levels similar to those of conventional food-grade surfactants in diverse oil systems. These results suggest that GJ1-derived biosurfactant may serve as a functional emulsifier in food systems. However, the emulsification stability evaluated in this study was limited to a relatively short timeframe. Therefore, the present findings should be interpreted as an initial assessment of emulsifying performance, and further studies are required to evaluate long-term storage stability under conditions relevant to food systems.

**Fig. 3:**
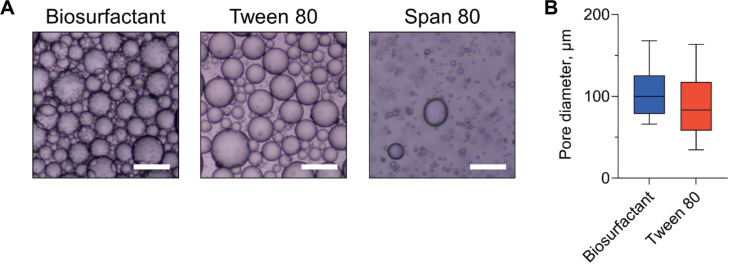
Microstructural characteristics of emulsions stabilized with the *Bacillus velezensis* GJ1-derived biosurfactant and commercial surfactants. (**A**) Optical micrographs of emulsions prepared using *B. velezensis* GJ1-derived biosurfactant, Tween 80, and Span 80 with sunflower oil after 24 h of storage. Scale bars represent 200 μm. (**B**) Comparison of droplet size distributions for emulsions stabilized with *B. velezensis* GJ1-derived biosurfactant and Tween 80 using sunflower oil after 24 h, determined by image-based analysis. Box plots represent the distribution of measured droplet diameters.

To further elucidate the emulsification behavior observed in EI measurements, the microstructural characteristics of emulsions stabilized with the *B. velezensis* GJ1-derived biosurfactant were examined and compared with those prepared using Tween 80 and Span 80 (Fig. 3A). Optical micrographs revealed that emulsions stabilized with the biosurfactant and Tween 80 formed densely packed and well-dispersed oil droplets in sunflower oil, resulting in continuous emulsion structures with broadly similar visual characteristics. These microstructural features are consistent with those reported for biosurfactant-stabilized emulsions in previous studies [38]. In contrast, emulsions prepared with Span 80 showed a distinctly different microstructural appearance, characterized by a sparse distribution of droplets and the presence of fewer, relatively large oil domains. Such microstructural features are consistent with previous observations of Span 80-stabilized emulsions in vegetable oil systems, where non-uniform droplet dispersion and heterogeneous emulsion structures have been reported [39]. Quantitative image-based analysis was subsequently used to assess droplet-level characteristics of emulsions stabilized with the biosurfactant and Tween 80 (Fig. 3B). The average droplet diameter of biosurfactant-stabilized emulsions was 104.4 ± 29.2 μm, whereas emulsions prepared with Tween 80 exhibited a slightly smaller mean droplet diameter of 84.1 ± 34.6 μm. Despite this difference in average droplet size, the droplet size distributions of the two emulsions overlapped substantially, indicating comparable microstructural organization at the droplet scale. Overall, the microstructural analysis supports the EI-based results, demonstrating that emulsions stabilized with the *B. velezensis* GJ1-derived biosurfactant exhibit droplet dispersion and microstructural characteristics comparable to those formed using Tween 80, a widely used food-grade emulsifier. Together, these findings indicate that the GJ1-derived biosurfactant is capable of forming oil-in-water emulsions with microstructural features similar to those of conventional food-grade surfactants.

### Antibacterial Activity of the *B. velezensis* GJ1 Biosurfactant against Foodborne Pathogen *in vitro* and in a Sausage-based Food Model

**Fig. 4:**
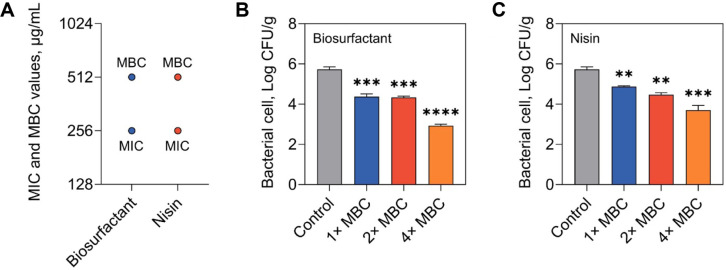
*In vitro* and sausage model antibacterial activity of the *Bacillus velezensis* GJ1-derived biosurfactant and nisin against *Escherichia coli*. (**A**) Minimum inhibitory concentration (MIC) and minimum bactericidal concentration (MBC) values determined by broth microdilution assay (*n* = 3). Reduction of *E. coli* populations in a sausage model system following treatment with (**B**) the biosurfactant and (**C**) nisin at 1×, 2×, and 4× MBC (*n* = 3). Bars represent mean ± standard deviation. Asterisks indicate significant differences compared with the untreated control (**p < 0.01, ***p < 0.001, and ****p < 0.0001).

To assess the antibacterial potential of the *B. velezensis* GJ1-derived biosurfactant prior to its application in food systems, its MIC and MBC were determined against the representative foodborne pathogen *E. coli* (Fig. 4A). The biosurfactant exhibited an MIC value of 256 μg/mL and an MBC value of 512 μg/mL against *E. coli*. In comparison, nisin showed identical MIC and MBC values of 256 μg/mL and 512 μg/mL, respectively. Similar or higher MIC and MBC values have been reported for *Bacillus*-derived biosurfactants, with antibacterial activity varying depending on bacterial species and assay conditions [40-43]. This trend has been attributed to the interaction of biosurfactants with bacterial cell membranes, leading to membrane destabilization and increased permeability, as reported in previous studies [14, 44]. Overall, these results suggest that the *B. velezensis* GJ1-derived biosurfactant demonstrates antibacterial activity comparable to that of nisin against *E. coli*, supporting its potential as a food-grade antimicrobial agent and providing a basis for subsequent evaluation in a sausage model system.

Subsequently, the antibacterial efficacy of the *B. velezensis* GJ1-derived biosurfactant was evaluated in a sausage-based food model inoculated with *E. coli* (Fig. 4B and 4C). This experiment was designed as a short-term antibacterial assessment in a food matrix to evaluate the immediate inhibitory effect of the biosurfactant under refrigerated conditions. The untreated control samples exhibited bacterial populations of 5.73 ± 0.13 log CFU/g after storage. Application of the biosurfactant led to a concentration-dependent decrease in viable cells. At 1× and 2× MBC, *E. coli* counts declined to 4.38 ± 0.19 and 4.34 ± 0.08 log CFU/g, respectively, whereas treatment at 4× MBC further reduced the population to 2.93 ± 0.20 log CFU/g, corresponding to an approximately 2.8 log reduction relative to the control. Nisin treatment also decreased bacterial populations, yielding counts of 4.87 ± 0.12, 4.30 ± 0.15, and 3.71 ± 0.18 log CFU/g at 1×, 2×, and 4× MBC, respectively. Notably, at the highest concentration tested, the biosurfactant achieved a greater reduction in *E. coli* than nisin under identical conditions. Nisin was included as a reference food-grade antimicrobial; however, it is generally known to be more effective against Gram-positive bacteria [45], and therefore this comparison should be interpreted with caution. The persistence of antibacterial activity within the sausage matrix indicates that the GJ1-derived biosurfactant retains antimicrobial efficacy in a complex food environment. Because food components can influence the distribution and activity of antimicrobial agents [17], demonstration of efficacy under matrix conditions is particularly relevant. Similar applications of biosurfactants in food systems have been reported, including inhibition of *Staphylococcus aureus* in pork using a *B. subtilis* SOPC5-derived biosurfactant [46] and suppression of *Listeria monocytogenes* in Chinese kale by a *Lactobacillus plantarum* MGL-8-derived biosurfactant [47]. Consistent with these reports, the present findings confirm that the GJ1-derived biosurfactant can effectively control *E. coli* in a processed meat model, supporting its potential use as a multifunctional ingredient for food safety enhancement.

## Conclusion

This study evaluated the potential of a biosurfactant derived from *B. velezensis* GJ1 as a multifunctional ingredient for food applications. WGS confirmed the presence of key lipopeptide biosynthetic gene clusters, including the surfactin and iturin operons. Notably, the genome was confirmed to lack major virulence factors and genes associated with foodborne toxins, including hemolysin BL, non-hemolytic enterotoxin, and the emetic toxin cereulide, supporting the potential safety of this strain at the genomic level. The biosurfactant demonstrated emulsifying performance comparable to commonly used food-grade surfactants such as Tween 80 and Span 80 across various vegetable oils, and microstructural analysis further confirmed the formation of stable oil-in-water emulsions with droplet characteristics similar to those of conventional emulsifiers. In addition to its emulsifying properties, the biosurfactant exhibited antibacterial activity against *E. coli* with MIC and MBC values comparable to those of nisin. Furthermore, significant reductions in *E. coli* populations were observed in a sausage-based food model, indicating that the antibacterial activity of the biosurfactant was retained within a complex food matrix. Collectively, these findings demonstrate that the biosurfactant from *B. velezensis* GJ1 possesses both emulsifying and antibacterial functions, highlighting its potential as a natural alternative to synthetic surfactants and conventional antimicrobials in food systems.
